# Aggressive Papillary Thyroid Carcinoma Presenting with Metastasis to the Pancreas

**DOI:** 10.1155/2022/5355419

**Published:** 2022-01-20

**Authors:** Firas Warda, Sam Ho, Enoch Kuo, Dinesh Rao, Marilu Jurado-Flores

**Affiliations:** ^1^Division of Endocrinology, Diabetes, and Metabolism, University of Florida College of Medicine-Jacksonville, Jacksonville, FL 32209, USA; ^2^Department of Medicine, University of Florida College of Medicine-Jacksonville, Jacksonville, FL 32209, USA; ^3^Department of Pathology, Immunology and Laboratory Medicine, University of Florida College of Medicine-Gainesville, Gainesville, FL 32610, USA; ^4^Department of Radiology, University of Florida College of Medicine-Jacksonville, Jacksonville, FL 32209, USA

## Abstract

Papillary thyroid cancer is the most common type of thyroid cancer. Aggressive forms tend to metastasize to the lungs and bones, but the abdomen is a rare site of metastasis. We present a 46-year-old male patient who presented with a neck mass associated with shortness of breath and hemoptysis. He was found to have a large thyroid mass on imaging. He underwent a total thyroidectomy with bilateral neck dissection, with pathology showing a multifocal tall cell variant of papillary thyroid carcinoma with lymphovascular invasion in both thyroid lobes. Due to recurrent findings of residual thyroid tissue on whole-body scan imaging, the patient underwent radioactive iodine ablation therapy twice, with poor response to therapy, suggested by persistently elevated thyroglobulin levels. However, the residual tissue responded to external beam radiation. After the initial response to radiation, thyroglobulin was noted to have increased again, prompting a PET-CT after administration of recombinant TSH. PET showed a focal area of increased uptake in the head of the pancreas. The patient underwent the Whipple procedure for resection of the metastasis. Pathology showed papillary thyroid carcinoma with strong and diffuse staining for TTF-1 and thyroglobulin. The patient was started on lenvatinib in the postoperative period and is currently tolerating treatment well with evidence of decreasing thyroglobulin levels. Intra-abdominal metastasis from a thyroid malignancy source is quite rare and can be challenging as far as diagnosis and treatment. Surgical resection can be curative and can be followed by radioactive iodine ablation therapy if cancer cells show avidity. Tyrosine kinase inhibitors can be used in refractory disease. New research is being conducted on new agents that can reverse the resistance to radioactive iodine therapy.

## 1. Introduction 

Papillary thyroid cancer (PTC) continues to be the most common thyroid cancer with the incidence being on the rise likely due to increased use of imaging modalities and ease of detection [1]. Since 1975, the incidence of thyroid cancer has tripled, and the entire increase has essentially been attributed to PTC [2]. Unfortunately, up to 10% of patients with papillary and follicular thyroid carcinomas present with distant metastasis [3]. A study done by Durante et al. showed that the 10-year survival in patients with well-differentiated thyroid carcinoma is reduced from 80–95% to 14% in patients older than 40 years of age with macronodular lung metastases or multiple bone metastasis [4]. Thyroid cancer metastasis to the gastrointestinal tract is unusual and only accounts for 0.5–1% of all distant metastases [5]. PTC metastasis to the pancreas is extremely rare [6], with only a dozen of cases reported in the literature to the best of our knowledge. Here, we present a patient with an initial diagnosis of mpT4a, N1b papillary thyroid cancer with pancreatic metastasis detected on positron emission tomography-computed tomography (PET-CT) and confirmed after undergoing radical pancreaticoduodenectomy, close to three years after initial diagnosis.

## 2. Case Presentation

**Figure 1:**
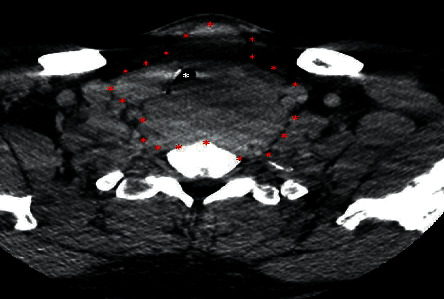
Enhanced axial computed tomography image at the level of the upper trachea demonstrating a large thyroid mass (red asterisks) resulting in compression and right lateral displacement of the trachea (white asterisk).

**Figure 2:**
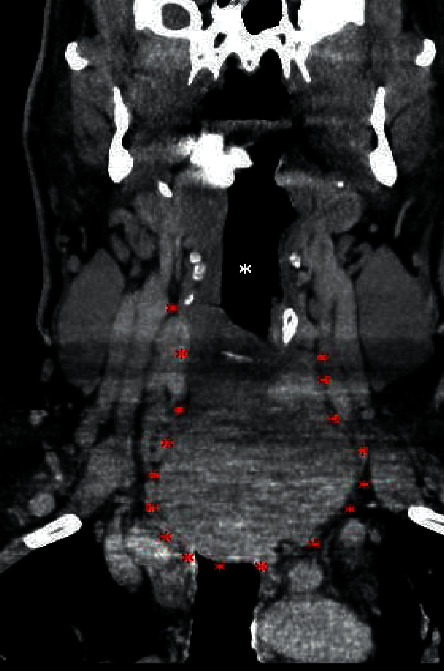
Enhanced coronal computed tomography image demonstrating a large thyroid mass (red asterisk) with invasion of the superior mediastinum. The airway (white asterisk) is displaced outside the field of view of this image.

**Figure 3:**
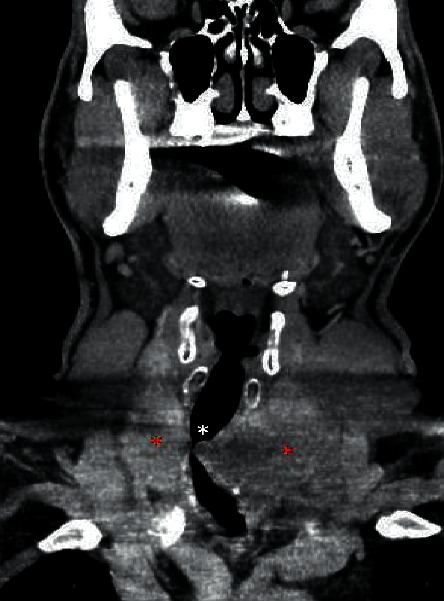
Enhanced coronal computed tomography image demonstrating severe narrowing of the trachea (white asterisk) by bilateral thyroid lobe tumor (red asterisks).

The patient is a 46-year-old male who complained of a neck mass that he noticed 4 months prior to presentation, associated with hoarseness, shortness of breath, and occasional hemoptysis. Physical exam showed a 5 cm mass in the anterior neck fixed to the trachea with extension to the left thyroid lobe and hoarseness of voice. Subsequent pharyngoscopy showed tracheal narrowing and paralysis of the left vocal cord. Computed tomography (CT) scan of the neck soft tissue showed a large thyroid mass arising from the left thyroid gland (Figure 1) with extension into the posterior upper mediastinum (Figure 2), paravertebral space, and left tracheoesophageal region with severe narrowing of the trachea (Figure 3), and right tracheoesophageal lymphadenopathy. He underwent total thyroidectomy, along with central, left-sided, and right tracheoesophageal lymph node dissection. Intraoperatively, left recurrent laryngeal nerve invasion of the tumor was noted in addition to circumferential encasement of the trachea. The postoperative period was complicated by severe and persistent hypocalcemia, requiring IV and oral calcium loading and initiation of calcitriol. Pathology showed a multifocal bilateral tall cell variant of papillary thyroid carcinoma, 6.5 cm in the greatest dimension, with perineural and lymphovascular invasion, and metastasis to one left-sided lymph node. The posterior resection margin of the left thyroid lobe and isthmus was involved by the papillary carcinoma.

**Figure 4:**
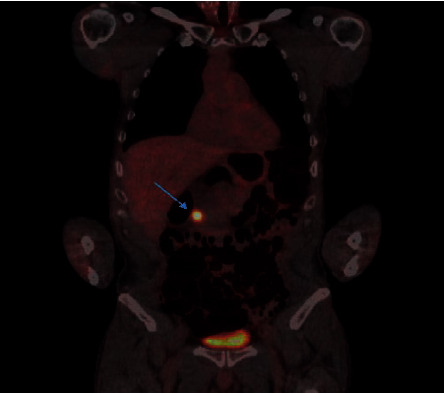
Positron emission tomography-computed tomography scan showing a focus of intense activity corresponding to the area of the pancreatic head (blue arrow) without a clear anatomical correlate.

**Figure 5:**
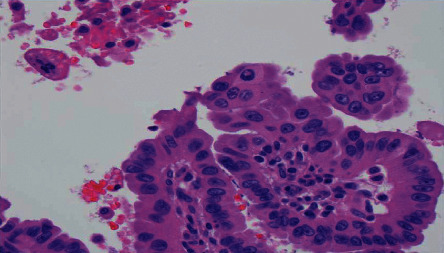
Hematoxylin and eosin stain. The tumor consisted of papillae lined by cells with the hallmark nuclear features of papillary thyroid cancer, such as nuclear grooves and pseudoinclusions. No psammoma bodies were seen. Overall, the histologic appearance was consistent with metastatic papillary thyroid carcinoma.

**Figure 6:**
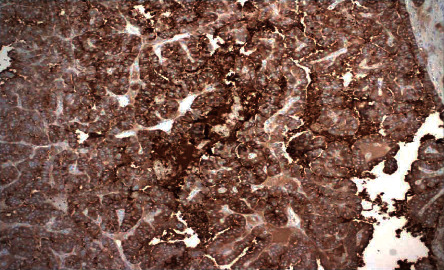
Immunohistochemistry for thyroglobulin was diffusely positive in tumor cells. This pattern confirms the tumor's thyroid origin.

Following thyroid hormone withdrawal, a whole-body scan along with single-photon emission computed tomography (SPECT) showed a focal area of increased radiotracer activity within the right thyroid bed representing residual thyroid tissue. There was a subtle area of increased uptake in the left thyroid bed representing residual malignancy. No evidence of distant metastatic disease was found. The patient was treated with 200 *μ*Ci of ^131^I radioactive iodine, followed by a postablative whole-body scan, which showed a focal area of increased radiotracer uptake at the left thyroid bed and prominent soft tissue indentation on the left posterolateral trachea, likely representing residual neoplasm. Four months later, the patient presented with hemoptysis and shortness of breath, for which CT of the chest was done and showed a large thyroid mass causing significant tracheal and esophageal luminal narrowing as well as esophageal leftward displacement. Postwithdrawal whole-body scan showed only minimal increase in radiotracer activity within the thyroid bed. Following a second treatment with 200 *μ*Ci of ^131^I radioactive iodine, postablative whole-body scan showed relatively minimal uptake within the tumor, suggesting a poor response to therapy. Unfortunately, a tracheostomy tube and percutaneous endoscopic gastrostomy (PEG) tube had to be placed for airway protection and feeding, respectively. The patient underwent 35 treatments of external beam radiation (EBR), after which thyroglobulin levels decreased to 1.3 ng/mL from 270 ng/mL, and the tumor size partially decreased. The patient was monitored with neck ultrasonography every 3–6 months, which repeatedly showed no residual thyroid tissue or suspicious lymphadenopathy. Approximately 3 years after diagnosis, the thyroglobulin level increased to 4.1 ng/mL. PET-CT scan showed a focus of intense activity corresponding to the area of the pancreatic head without a clear anatomical correlate (Figure 4). Magnetic resonance imaging (MRI) with contrast could not be done at the time as the patient was suffering from a contrast-induced acute kidney injury. Due to esophageal stenosis, endoscopic ultrasound could not be performed. After discussion with the surgical oncology team and patient, he underwent radical pancreaticoduodenectomy and placement of a gastrostomy-jejunostomy tube. Pathology showed metastatic papillary thyroid carcinoma involving the pancreas (Figure 5), 3.0 cm in the greatest dimension, with the tumor showing strong and diffuse nuclear staining for TTF-1 and cytoplasmic staining for thyroglobulin (Figure 6). Sixteen peripancreatic lymph nodes and one pericystic lymph node were removed during the operation and found to be negative for carcinoma. Genetic analysis was performed on the pancreatic lesion using Guardant 360 assay (Guardant Health Inc., Redwood City, CA). Gene amplification was detected in BRAF, KRAS, EGFR, and PIK3CA genes. Alteration in the promoter region of the TERT gene was also reported. Finally, fusion events involving RET, NTRK1, NTRK2, NTRK3, BRAF, and EGFR genes were reported as well. The patient was assessed by a medical oncologist, who started the patient on lenvatinib. The latest thyroglobulin level was 2.9 ng/mL. Unfortunately, lenvatinib treatment caused TSH to increase drastically, for which thyroid hormone dose was increased. The patient suffered from debilitating depression secondary to his medical condition, with notable and significant weight loss, prompting palliative care team involvement for goals of care discussion.

## 3. Discussion

While primary pancreatic tumors make up most pancreatic masses, metastatic lesions can be found in the pancreas, mainly from lung and renal primary malignancies [7]. Metastasis from PTC occurs in up to 34% of patients on follow-up after diagnosis and initial treatment, but it is usually local and rarely distant, with the liver being the most common site of metastasis in the gastrointestinal tract [8]. Aside from our case, there are only 15 reports of PTC metastasis to the pancreas in the literature [9]. Mean age at presentation was roughly 56.5 years. Like our patient, all reported cases had aggressive PTC on initial diagnosis, and mutations in the BRAFV600E gene were commonly found [8, 10–12]. Tall cell variant of PTC, which is known to be more aggressive in nature, was reported in only 3 patients in addition to our patient [6, 10, 13]. However, many cases showed nonavidity to radioactive iodine, which is known to be a poor prognostic factor as the loss of the sodium-iodine symporter is a sign of dedifferentiation [6, 8, 11–14]. It is well known that differentiated thyroid cancer has a female predominance, but strikingly, 11 of the reported cases including our patient were males. Most patients with pancreatic metastasis have undergone surgical resection, followed by tyrosine kinase inhibitor (TKI) therapy in only 3 cases [8, 9, 12]. Histologically, almost all cases reported positive staining for TTF-1 and thyroglobulin, except for one case [9]. Unique to two cases was the positive staining for Ki67 [9, 15]. In terms of location, the head of the pancreas was the most common location of metastasis, although 3 cases report multifocal disease in the tail and the body [15–17]. Lastly, the timing of presentation after primary tumor diagnosis varied widely between cases, starting as early as one month and as late as thirteen years. Only one case reported the metastatic disease at the same time as the primary cancer diagnosis [16].

The emergence of multiple targeted therapies has provided more hope in targeting metastatic thyroid cancer based on gene mutations that it may have. Some of the important gene mutations to screen for in thyroid cancer include BRAFV600E, RAS, RET, telomerase reverse transcriptase (TERT), and neurotrophic tyrosine kinase (NTRK) [18]. Depending on the gene mutation present, therapy can be specific for each patient.

Gene expression classifier (GEC) advancements have made it easier to detect such mutations at the time of fine-needle aspiration performance, which has been helpful in further stratifying patients with indeterminant nodules. This has reduced the number of patients that undergo surgery for such nodules given the high sensitivity of such tests [19].

As some aggressive forms of thyroid cancer become refractory to radioactive iodine, some agents were studied to determine whether they can resensitize thyroid cancer to radioactive iodine. Of interest, selumetinib (AZD6244), which is a mitogen-activated protein kinase (MAPK) inhibitor, was studied by Ho et al. in 2013 and found to produce a clinically significant increase in iodine uptake in a subgroup of patients with iodine-refractory thyroid cancer with RAS and BRAF gene mutations [18]. Dabrafenib (GSK2118436) was also studied by Rothenberg et al. in 2015 and showed benefit in redifferentiation of thyroid cancer in patients with BRAF gene mutation [20].

In our patient, metastasis developed despite TSH suppression, and surgical resection of the pancreatic metastasis was thought to be of benefit given it was the only site detected on the PET scan. Extensive discussion needs to be held with patients regarding future targeted therapy if any of the gene mutations return positive as such therapy is expected to cause a lengthy list of side effects.

## 4. Conclusion

We present an unfortunate case of aggressive primary thyroid cancer that has metastasized to the pancreas, a rare site of abdominal metastasis. Few points can be learnt from this case and previous similar cases:

- Despite the rarity of abdominal metastasis from primary thyroid cancer, it should always be considered as a possibility in a patient with rising thyroglobulin levels, especially with a history of high-risk features.
- Developing ultrasensitive thyroglobulin assays that can detect even minimal serum levels can aid in early detection and prompt evaluation [21].
- Discussion should be held with the patient regarding treatment options, including surgical interventions, medical therapy, radiation therapy, or combination of more than one.
- Further studies (and possibly updated guidelines) should look further at the appropriate frequency of biochemical and radiological monitoring of patients with aggressive disease.
- Tyrosine kinase inhibitors continue to be the mainstay of medical treatment of aggressive papillary thyroid cancer that is refractory to radioactive iodine treatment. However, the long list of side effects must be discussed with patients, particularly the need for a higher dose of the thyroid hormone.
- Multidisciplinary team including endocrinology, medical oncology, radiation oncology, general surgery, and palliative care must be involved in the care of such patients.
- More studies need to be conducted regarding the psychosocial burdens of thyroid cancer recurrence and active persistent disease on patients.

## Figures and Tables

**Figure 7:**
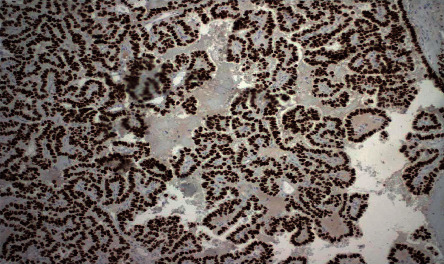
Immunohistochemistry for thyroid transcription factor-1 (TTF-1) was diffusely positive in tumor cells. This supports the tumor's thyroid origin.

## Data Availability

The data used to support the findings of this study are available upon request to the corresponding author.
